# Natural Killer Cells Modulation in Hematological Malignancies

**DOI:** 10.3389/fimmu.2013.00459

**Published:** 2013-12-19

**Authors:** Céline Baier, Aurore Fino, Carole Sanchez, Laure Farnault, Pascal Rihet, Brigitte Kahn-Perlès, Régis T. Costello

**Affiliations:** ^1^UMR1090 TAGC, INSERM, Marseille, France; ^2^UMR1090 TAGC, Aix-Marseille Université, Marseille, France; ^3^Service d’hématologie, APHM, Hôpital de la Conception, Marseille, France

**Keywords:** hematological malignancies, natural killer cells, natural cytotoxicity receptors, immune escape, immunotherapy

## Abstract

Hematological malignancies (HM) treatment improved over the last years resulting in increased achievement of complete or partial remission, but unfortunately high relapse rates are still observed, due to remaining minimal residual disease. Therefore, sustainment of long-term remission is crucial, using either drug maintenance treatment or by boosting or prolonging an immune response. Immune system has a key role in tumor surveillance. Nonetheless, tumor-cells evade the specific T-lymphocyte mediated immune surveillance using many mechanisms but especially by the down-regulation of the expression of HLA class I antigens. In theory, these tumor-cells lacking normal expression of HLA class I molecules should be destroyed by natural killer (NK) cells, according to the missing-self hypothesis. NK cells, at the frontier of innate and adaptive immune system, have a central role in tumor-cells surveillance as demonstrated in the setting of allogenic stem cell transplantation. Nevertheless, tumors develop various mechanisms to escape from NK innate immune pressure. Abnormal NK cytolytic functions have been described in many HM. We present here various mechanisms involved in the escape of HM from NK-cell surveillance, i.e., NK-cells quantitative and qualitative abnormalities.

## Introduction

**Figure 1:**
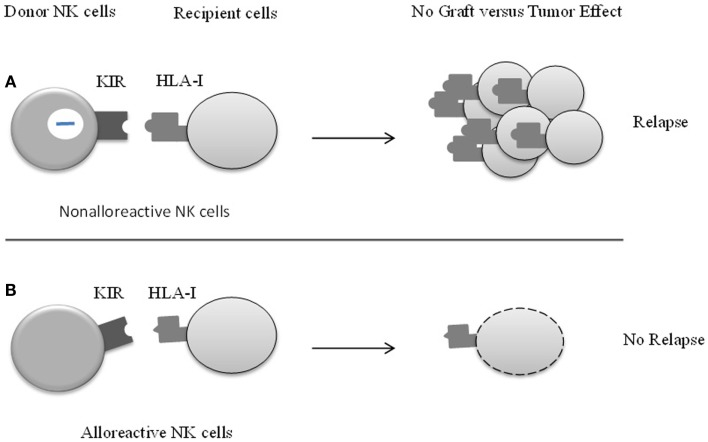
**The effect of KIR/HLA class I mismatch in patients undergoing allogenic stem cells transplantation (HSTC)**.The main challenge in HSTC is to keep the balance between conserving the graft versus leukemia (GvL) effect on the one hand and preventing risk of relapse on the other. **(A)** Killer cell immunoglobulin-like receptor (KIR) of donors NK cells is engaged by corresponding HLA ligand, which inhibits NK-cell function. Donor NK cells are non-alloreactive and do not kill recipient’s blasts, which leads to the relapse of patient. **(B)** The concept and recognition of KIR-ligand incompatibility (also known as KIR-ligand mismatch) has important implications. KIR-mismatch is an independent predictor of survival in patients. Graft versus tumor NK-cell alloreactivity reduces the risk of leukemia relapse, and markedly improves event-free survival.

The natural killer (NK) cells are central players in innate immunity particularly regarding the surveillance against malignant tumors (1, 2). NK role in tumor-cells clearance is proved by allogenic stem cells transplantation, since a better engraftment and a low relapse rate are observed when the graft NK inhibitory receptors mismatch with recipient HLA molecules (3) (Figure 1). The triggering event of NK-cells activation and killing of target cells results from a balance between activating and inhibitory signals sent by membrane receptors that either enhance or block the NK-mediated cytotoxicity (4). Inhibitory signals arise from interaction between HLA-specific inhibitory receptors, as the killer immunoglobulin-like receptors (KIR), NK group protein 2A (NKG2A), or Immunoglobulin-like transcript 2 (ILT-2) with HLA class I molecules, whereas the absence or abnormal expression of the later molecules induces NK-cells cytotoxicity (5). The down-regulation of HLA class I molecules is an immune escape mechanism frequently used by tumor cells (6) that, accordingly, should not be recognized by the T-lymphocyte receptor (TCR). The absence of normal HLA class I molecule on tumor cells should lead to NK-cells activation, more efficiently when co-stimulatory molecules and ligands for NK activating receptor are present at tumor cell surface. The natural cytotoxicity receptors (NCR) NKp46/NCR1, NKp30/NCR3, and NKp44/NCR2 (7), NKG2D, DNAM-1 and also co-receptors such as 2B4/CD244 and NTBA, play a central role in NK activation. Once activated, NK lymphocytes kill tumor cells via FcgRIIIA (CD16) which can trigger antibody-dependent cellular cytotoxicity (ADCC) on encountering target cells opsonized with IgG, via the Fas/Fas-L pathway and via cytotoxic granules (perforin/granzyme) secretion (1, 8).

Defects in NK-cell cytotoxicity have been described in most hematological malignancies (HM) (9–12) (Table 1). Interestingly, tumor-cells develop various escape mechanisms to NK-cell surveillance and contribute to the dysfunction of NK-cell cytotoxicity (4).

**Table 1:** **Mechanisms of immune escape and drugs used with their effects on NK cells in hematological malignancies**.

Hematological malignancies	Principal mechanisms of escape	References	Principal class of drugs used	Effects of drugs on NK activation	References
MDS	NK-cell quantitative deficiency Elevated TNF	(13, 14, 49)	Demethylating agents IMIDs	Up-regulation of KIR and NKG2D ligands	(76, 81)
				NKp46 upregulation	
AML	Upregulation of HLA-I Decrease expression of NKp30, 2B4/CD244 Production of ROS	(9, 19, 39, 40, 54)	HDACIs Histamine dihydrochloride All-trans retinoic acid Monoclonal antibody (IPH2101)	Upregulation of MICA/B expression	(65, 67, 72, 73, 84, 85)
				Suppress ROS production	
				Promote NK-cells cytotoxicity	
CLL	Upregulation of HLA-I low level of MICA or ULBP	(18, 37)	Monoclonal antibody	Mediate NK-cells cytotoxicity	(88)
MM	Production of sMICA/sMICB Weak expression of CD16	(30, 31)	IMIDs Proteasome inhibitor IPH2101	NKp46 upregulation NKG2D ligand upregulation	(79, 82–84, 86)
CML	Production of sMICA and weak expression of NKG2D	(29)	Tyrosine kinase inhibitor	sMICA down-regulation and NKG2D upregulation	(29)
ALL	Low production of MICA/B and ULBP	(26, 27, 41)			
	Down regulation of HLA-A and HLA-Bw6	

*MDS, myelodysplastic syndrome; AML, acute myeloid leukemia; CLL, chronic lymphocytic leukemia; MM, multiple myeloma; CML, chronic myelogenous leukemia; ALL, acute lymphoblastic leukemia; s/MICA/B, soluble/stress-induced molecules human class I-like molecules A and B; ULBPs, UL-16 binding proteins; ROS, reactive oxygen species; KIR, killer immunoglobulin-like receptors; PDGF, platelet-derived growth factor; HDC, histamine dihydrochloride; IMIDs, immune-modulatory drugs; HDACIs, Histone deacetylase inhibitors*.

## NK-Cells Dysfunctions in Hematological Malignancies

### Quantitative abnormalities

The first mechanism explaining tumor escape is the quantitative abnormalities of NK cells. In myelodysplastic syndrome (MDS) patients, decreased NK-cell cytolytic functions correlate with a low number of circulating NK cells and a high level of sIL-2R (13, 14). In chronic myelogenous leukemia (CML), functional NK-cell deficiency can be reversed *in vitro* by IL-2 (15), but this effect is progressively lost while a progressive decrease in NK-cell number is observed (16). In Philadelphia (Ph1)-negative myeloproliferative syndrome (MPS), NK cytotoxic activity is decreased, mostly in idiopathic myelofibrosis (IMF) patients. The percentage of NK cells is decreased in IMF and increased in polycythemia vera (PV) (17). We have confirmed that the percentage and absolute number of NK cells are significantly increased in PV, but we failed to detect any abnormalities in the expression of activating NK-cell receptors or cytotoxic functions (personal data, C. Sanchez). An increase in the total number of NK cells in the peripheral blood has also been described in chronic lymphocytic leukemia (CLL) but still associated with defective cytolytic functions (18).

### Altered activating receptors profiles

In acute myeloid leukemia (AML) the down-regulation of activating receptors NKp30/NCR3 and NKp46/NCR1 correlates with defective NK-cell cytotoxicity and poor leukemia prognosis (9, 19). In patients attaining complete remission (CR) after chemotherapy, NKp46/NCR1 expression returns to normal levels while patients who do not achieve CR or who relapse maintain abnormal NCR expression (9, 19). The defect in NCRs expression could be potentiated by the low expression of NCR and NKG2D ligands by leukemic cells (20–22). Down-regulation of the NK activating receptors/co-receptor DNAM-1, 2B4/CD244, and CD94/NKG2C have also been reported in AML (23, 24). Leukemic blasts that express DNAM-1 ligands induce DNAM-1 down-regulation at the NK-cell surface (25), thus impending NK-cell functions.

In acute lymphoblastic leukemia (ALL), expression of the NKG2D activating receptor ligands MICA/B was only observed in NK sensitive T-ALL cell line, while NK-resistant B-ALLs did not express detectable amounts of MICA/B (26). Deficient engagement of other activating receptors may also contribute to ALL resistance to NK lysis, since B-ALL cells lose or express low levels of several other NK activating ligands such as UL-16 binding proteins (ULBPs), PVR (polio virus receptor, CD155), Nectin-2 (CD112), or CD48 (27).

In MDS, a pre-leukemic stage, Epling-Brunette et al. (13) have shown that expression of NKp30/NCR3 and NKG2D was decreased, in contrast with the data of Kiladjian et al. (28); this discrepancy could be related to the heterogeneity of MDS patients.

In CML patients, Boissel et al. (29) reported high serum sMICA levels and weak NKG2D expression on NK cells, that correlate with low NK-cell cytotoxicity capacities. Imatinib mesylate, the first inhibitor of tyrosine kinase used in CML, increases NKG2D expression and decreases MICA protein production and release, thus contributing to normal NK cytotoxicity through the restoration of a functional NKG2D signaling (29).

Monoclonal gammopathy of undetermined significance (MGUS) is a common disorder of aging and a precursor lesion to multiple myeloma (MM). In MGUS, tumor-cells express high levels of MICA, whereas low levels of sMICA are detected in peripheral blood (30). This explains the capacities of NK cells to kill MGUS tumor cells by interaction between MICA and NKG2D. Conversely, MM patients present high plasma level of sMICA while tumor-cells express low level of MICA, thus impending NK stimulation via NKG2D (31). This reveals that the alterations in the NKG2D pathway signaling are associated with the progression from MGUS to MM (30, 32). In peripheral blood from patients with MM a normal expression of the NCRs and NKG2D is observed, while 2B4/CD244 and the low-affinity Ig- Fc receptor CD16 display significantly weaker expression in comparison with healthy donors (31). Nonetheless, when NK are studied at the site of tumor location, i.e., bone marrow, (33) a drastic down-regulation of three major activating NK receptors (NKp30/NCR3, NKG2D, and 2B4/CD244) is observed in comparison with bone marrow from healthy donors (34). This suggests that some NK abnormalities may be underestimated if only peripheral blood is studied.

In CLL, NK cells have weak cytotoxic functions which can be restored by stimulation with recombinant IFN-γ or IL-2 (35). We failed to detect a difference in NCR expression between patients and age-matched healthy donors (36), but we found a correlation with abnormal activating molecule expression and poor prognosis factors. In CLL decreased NK functions could be explained in part by absent or low level of MICA or ULBP (18, 37) at CLL lymphocyte cell surface, thus impeding NKG2D engagement.

### Abnormal KIRs phenotype and inhibitory molecules

Killer immunoglobulin-like receptor-mismatch in allogenic stem cells transplantation improves the disease-free survival in AML (38). In fact, NK-cell cytotoxicity is down-regulated by the engagement of HLA-specific inhibitory receptors, including KIRs and CD94 and NKG2A/B heterodimers. The analysis of KIR phenotype in AML patients shows that the frequency of particular inhibitory KIRs in association with their putative HLA class I ligands is significantly increased compared to healthy donors (10, 39, 40). This supports the hypothesis that AML blasts escape from immune surveillance according to the dominance of inhibitory over activating KIR signals. In ALL, the resistance of B-cell precursors to cytotoxicity is explained by the interaction between HLA-G and KIR2DL4 (26). Demanet et al. (41) have observed in ALL and CLL a selective down-regulation of HLA-A and HLA-Bw6 associated with HLA-Bw4 preservation, which provided an escape mechanism from NK-cell immune surveillance. Maki et al. (18) reported in CLL cells an increased expression of HLA-G1, a class I molecule that engages NK-cell inhibitory molecules and which has for ligand p49/KIR2DL4/CD158d (expressed on NK cells and a fraction of T cells), ILT-2 [expressed on NK, T, B cells, dendritic cells (DC), and monocytes], and ILT-4 [expressed on antigen-presenting cells (APC)] (42).

## Tumor Environment and Role of Cytokines

One of the strategies used by tumor cells having an effect on NK-cell function is the production of inhibitory molecules, which decrease NK-cells number and inhibit NK-cell activation. Increasing evidence supports the role of the tumor microenvironment in conferring drug resistance, a major cause of relapse and incurability of cancers. Tumor microenvironment includes tumor-cells contact and interaction, but also production of soluble factors, which provide signals for tumor growth and survival or inhibition of NK.

### Role of cytokines and cellular ligands

Several cytokines decrease NK-cell activation and cytotoxicity, such as the Transforming Growth Factor beta (TGF-β). High circulating TGF-β level correlates with poor prognosis in acute leukemia (43) and is linked to reduced NK-cell activity with reduced expression of NKp30/NCR3 and NKG2D (44). TGF-β antagonizes IL-15, a cytokine that induces NK-cell proliferation and activation. Thus, TGF-β inhibits the expression of both NK-cell activation receptor molecules and components of the cytotoxic apparatus (45, 46). Several studies have also underlined that low INF-γ producing capacity of NK cells was correlated with loss of NK-cell cytotoxicity (47, 48).

A physiologic concentration of platelet-derived growth factor (PDGF) significantly inhibits human NK-cell cytotoxicity. Patients suffering from IMF and essential thrombocythemia have significantly elevated circulating levels of plasma PDGF. Pretreatment of normal NK cells with concentrated PDGF-containing platelet-poor plasma from patients with these diseases significantly inhibits NK cytotoxicity. This inhibitory effect is reversed by neutralization of plasma PDGF with anti-PDGF (17, 49). All these data suggest that PDGF is probably a key factor of NK functional deficiency in MPS.

Interaction between tumor cells and NK impairs NK-cell-mediated cytotoxicity and thus induces tolerance to tumor invasion. Up-regulation of the immunosuppressive cell surface glycoprotein CD200 and of soluble GITRL (glucocorticoid-induced TNFR related protein ligand) on AML cells specifically compromises NK-cell anti-tumor responses (50, 51) and is a poor prognosis factor. AML cells exert direct immunosuppressive effects on NK cells mediated by immunosuppressive ligands or soluble factors and induce regulatory T lymphocytes (Treg) that weaken NK-cell responses (52). NK cells can also interact with DC leading to activation of Treg and inhibition of NK cells (53). Recently, ligand of NKp44/NCR2 (NKp44L) was identified as an isoform of mixed-lineage leukemia-5 (MLL5) (54). This ligand is not detectable in the normal tissues but is present in hematopoietic, non-hematopoietic tumor and transformed cells. The expression of MLL5 is a good prognosis factor in AML (55). Thus we can speculate that the prognostic value of MLL5 expression is linked to its capacity to activate anti-leukemia NK cells.

### Reactive oxygen species

Non-malignant phagocytic cells down-modulate lymphocyte functions by producing and releasing NADPH oxidase-derived reactive oxygen species (ROS) (56). Monocytic and myelo-monocytic (French-American-British classification M4/M5 subtypes) AML cells, but not cells from myeloblastic (FAB class M2) or immature (FAB class M1) AML, produce ROS via the NADPH oxidase component gp91phox, and trigger extensive apoptosis of NK cells *via* a poly-[ADPribose] polymerase-1 dependent pathway, together with a down-regulation of NKp46/NCR1. This suggests a novel mechanism of immune evasion in myelo-monocytic and monocytic AML (57).

### Material transfer during cell–cell contact

Tumor membrane-derived microvesicles (tMV) are important mediators of cell-to-cell communication. These circular membrane fragments are enriched in various bioactive molecules and directly stimulate cells as a kind of “signaling complex.” An important mode of communication between carcinoma cells and immune cells involves tMV, also known as exosomes, ectosomes, or microparticles. These microvesicles carry lipids, proteins, mRNAs, and microRNAs and travel short or long distances to deliver undegraded and undiluted material to other cells (58). Microvesicles present in AML patients’ sera contain TGF-β that down-regulates the expression of NKG2D and thus interfere with NK-cell activation. Nonetheless, IL-15 protects NK cells from adverse effects of tMV and could thus contribute to maintain their anti-tumor response (59). Baj-Krzyworzeka et al. (60) have observed that tMV carry mRNA of tumor cells and transfer some of them to monocytes and modify their activity. This type of mRNA transfer could participate to NK inactivation and tumor escape to innate immunity.

## Immunotherapy Approaches

Modulation of NK-cell function by down-regulation of receptors and/or ligand corresponds to an immune escape mechanism for tumors. Restoring the expression of activating receptors on NK cells, or corresponding ligands on cancer cells, is an effective approach to cancer immunotherapy in order to improve disease-free survival after therapy. Currently, in most AML patients, the induction treatment leads to CR, defined as microscopic disappearance of leukemic disease along with the return of normal hematopoiesis. However, many patients in CR relapse with poor prospects of long-term survival. One of developing immunotherapy that enhances NK-cell ability to kill tumor cells is the allogenic transplantation after chemotherapy. In AML, a recipient of haploidentical allogenic transplant with a NK HLA-specific receptor-mismatch is associated with a favorable prognosis because this increases the anti-leukemic graft reactivity (61, 62). Rapid recovery of NK cells after hematopoietic stem cell transplantation has been associated with a reduction in the rates of relapse and acute graft-versus-host disease (GvHD) (63). The transfer of NK cell from haploidentical origins into AML recipients is a potent immunotherapy intervention that is, unfortunately, associated with a significant transplant-related morbidity and mortality that limit its use (64).

The use of cytokines is another therapeutic approach to enhance NK-cell cytotoxicity. IL-2, IL-12, Il-15 (65), and IL-18 have been used in culture to increase cell cytotoxic prior to the injection of NK cells in cancer patients (66). Another strategy consists in restoring normal NCR expression since these molecules are pivotal for the anti-leukemia response. A phase III study in 320 AML patients has demonstrated that immunotherapy with histamine dihydrochloride (HDC) and IL-2 decreases and delays relapses in AML (67). HDC suppresses or inhibits ROS formation in mononuclear and polymorphonuclear myeloid cells. This prevents from oxygen radical-induced NK apoptosis, restores NK-cell capacity to respond to IL-2, and improves NK proliferation and production of immuno-stimulatory cytokines (56, 68–71). Moreover, in presence of HDC, cytotoxic functions of NK cells remain intact due to the preserved expression of the activating receptors NKG2D and NKp46/NCR1.

DNA methylation also has a key role in the control of gene activity in cancer cells. Two agents are currently used in MDS treatment: 5-azacytidine (Vidaza) and 5-aza-20-deoxycytidine (Decitabine) (72). These two hypomethylating agents up-regulate NKG2D ligands MICA/B leading to enhanced NK-cell cytotoxicity (73, 74). Conversely we have observed the down-regulation of 2B4/CD244 in NK from AML patients treated with 5-azacytidine, that could have an opposite effect, i.e., cytotoxicity down-regulation (Leclerq et al. personal data).

The immune-modulatory drugs (IMIDs) such as thalidomide and lenalidomide are used in MM and MDS treatment and have anti-angiogenic and anti-inflammatory properties. They also act as IMIDs by cytokine release and activating effector cells by enhancing ADCC and NK-cell cytotoxicity thanks to the up-regulation of NKp46/NCR1 (75–77).

Bortezomib is a proteasome inhibitor used in MM treatment. Physiologically, proteasome is involved in protein degradation. Its inhibition by the drug interferes with tumor growth and with innate immunity. Bortezomib is involved in the down-regulation of HLA I molecules and in the up-regulation of NKG2D, TRAIL, and DNAM ligands, thus leading to increased NK-cell cytotoxicity against plasma cell (78, 79).

Another attractive therapeutic approach consists in blocking the NK inhibitory receptors. A phase 1 trial has tested the IPH2101, a fully humanized IgG4 anti-KIR monoclonal antibody, in patients with relapsed/refractory AML (clinical trial registration number NCT01256073) and MM (clinical trial registration number NCT00999830). IPH2101 promotes immune complex formation and NK-cell cytotoxicity specifically against MM cell targets but not normal cells. No evidence of autoimmunity was observed. These findings suggest that IPH2101 is safe and tolerable and that this approach warrants further development in MM and AML (80–82) as we are waiting for clinical results.

Several studies revealed ADCC as one major mode of action of antibody-based therapeutics and stimulated more interest in how to mobilize, expand, and activate NK cells in humans (83). In CLL, the ADCC pathway via the Fc receptor (FcgRIIIa) CD16 at surface of NK cells is pivotal in the clinical effect of mAbs such as rituximab or ofatumumab which mediate ADCC by NK cells (84). Second generation mAb are designed in order to maximize both direct apoptosis and ADCC. The role of ADCC is underlined by better clinical responses to rituximab when NK cells expressed the high-affinity form of the FcgRIII (85, 86).

Bi-specific NK-cell engagers (BiKE) simultaneously bind CD16α and c-Met (a receptor overexpressed in many tumors) and thus may increase NK-cell ADCC (87).

Another type of therapeutic strategy consists in taking advantage from anti-cancer drugs properties (Table 1). Chemotherapy drugs can be separated by their ability to inhibit (such as vinblastine, chlorambucil, docetaxel) or enhance (such as asparaginase, bleomycin, doxorubicin) NK-cell-mediated killing of target cells (88, 89). In cancer, epigenetic changes are also involved in dysregulating NK-cell ligand expression. Histone deacetylase inhibitors (HDACIs) such as trichostatin, are epigenetic anti-cancer agents that modulate innate immunity by the regulation of expression of NKG2D or DNAM-1 ligands. Indeed, HDACIs increase expression of ligands of these two activating receptors, MICA/B or PVR and Nectin-2 respectively, on acute leukemia cells (90, 91). This suggests that epigenetic drugs make tumor cells more sensitive to NK-cell-mediated lysis (92). However, it has also been demonstrated that HDACIs suppress NK-cell cytotoxic activity by down-regulation of NKp30/NCR3, NKp46/NCR1 (93). Thus, the same drug can have contradictory effects on NK cells.

## Concluding Remarks

The more precise and exhaustive analysis of NK dysfunction in HM has opened the way to novel therapeutic strategies involving either specifically developed drugs/antibodies or innovative use of “old” drugs such as IMIDs. Due to the complexity of the immune response and the putative opposed effects of a drug on the various partners of the immune network, data obtained by *in vitro* experiments or *in vivo* in animal models have to be evaluated in clinically and biologically carefully monitored clinical trials.

## Conflict of Interest Statement

The authors declare that the research was conducted in the absence of any commercial or financial relationships that could be construed as a potential conflict of interest.
